# Differences in responses to the intracellular macrophage environment between *Mycobacterium bovis* BCG vaccine strains Moreau and Pasteur

**DOI:** 10.1590/0074-02760230070

**Published:** 2023-10-13

**Authors:** Paloma Rezende Corrêa, Marcos Gustavo Araujo Schwarz, Renata Monteiro Maia, Fátima Maria Figueroa Vergara, Milton Ozório Moraes, Leila Mendonça-Lima

**Affiliations:** 1 Fundação Oswaldo Cruz-Fiocruz, Instituto Oswaldo Cruz, Laboratório de Genômica Funcional e Bioinformática, Rio de Janeiro, RJ, Brasil; 2 Fundação Oswaldo Cruz-Fiocruz, Instituto Oswaldo Cruz, Laboratório de Biologia Molecular de Insetos, Rio de Janeiro, RJ, Brasil; 3 Fundação Oswaldo Cruz-Fiocruz, Instituto de Tecnologia em Fármacos, Laboratório de Farmacologia Aplicada, Farmanguinhos, Rio de Janeiro, RJ, Brasil; 4 Fundação Oswaldo Cruz-Fiocruz, Centro de Desenvolvimento Tecnológico em Saúde, Fiocruz, Rio de Janeiro, RJ, Brasil; 5 Fundação Oswaldo Cruz-Fiocruz, Instituto Oswaldo Cruz, Laboratório de Hanseníase, Rio de Janeiro, RJ, Brasil

**Keywords:** tuberculosis, BCG vaccine, *Mycobacterium bovis* BCG Moreau, intraphagosomal environment, THP-1 derived macrophages

## Abstract

**BACKGROUND:**

The Bacille Calmette-Guérin (BCG) vaccine comprises a family of strains with variable protective efficacy against pulmonary tuberculosis (TB) and leprosy, partly due to genetic differences between strains.

**OBJECTIVES:**

Previous data highlighting differences between the genomes and proteomic profiles of BCG strains Moreau and Pasteur led us to evaluate their behaviour in the macrophage microenvironment, capable of stimulating molecular responses that can impact the protective effect of the vaccine.

**METHODS:**

Strain infectivity, viability, co-localisation with acidified vesicles, macrophage secretion of IL-1 and MCP-1 and lipid droplet biogenesis were evaluated after infection.

**FINDINGS:**

We found that BCG Moreau is internalised more efficiently, with significantly better intracellular survival up to 96 h p.i., whereas more BCG Pasteur bacilli were found co-localised in acidified vesicles up to 6 h p.i. IL-1β and MCP-1 secretion and lipid droplet biogenesis by infected macrophages were more prominent in response to BCG Pasteur.

**MAIN CONCLUSION:**

Overall, our results show that, compared to Pasteur, BCG Moreau has increased fitness and better endurance in the harsh intracellular environment, also regulating anti-microbial responses (lower IL-1b and MCP-1). These findings contribute to the understanding of the physiology of BCG Moreau and Pasteur in response to the intraphagosomal environment in a THP-1 macrophage model.

Tuberculosis (TB) is a serious global health problem and occupied the first place in the world ranking of deaths by infectious diseases since 2015, being recently surpassed by coronavirus disease 19 (COVID-19).^1^ Despite considerable efforts to control the disease, 10 million new cases were observed in 2020 with 1.5 million deaths. Most of the affected population is distributed in developing countries, due to low socioeconomic conditions.^1^

The process of infection of the host by *Mycobacterium tuberculosis* (*Mtb*) occurs in a sequence of well-established events. Transmission occurs through inhalation of aerosols containing the bacillus, which is phagocytised by macrophages resident in the lung.^2^ The recognition and phagocytosis of *Mtb* by macrophages induces the expression of a variety of genes that control the immune function of these phagocytes, aiming to eliminate the pathogen. For this, several microbicidal mechanisms are employed, among them the production of reactive oxygen species (ROS) and reactive nitrogen intermediates (RNI), in addition to phagosomal acidification and the secretion of cytokines and chemokines.^3^

The phagosomal maturation process occurs through successive fusions and fissions with intracellular vesicles, proceeding from the initial phagosomes to late and then phagolysosomes. The phagolysosome is characterised by an acidic pH (4 - 4.5) and high hydrolase activity, causing the death of mycobacteria.^4^ ^,^ ^5^ Bacteria contained in phagosomes, such as *Mtb* and *M. bovis* Bacille Calmette-Guérin (BCG), not only interact with vesicles of various origins during maturation, but also with lipid storage compartments, such as lipid droplets.^6^ ^,^ ^7^

Bacille Calmette-Guérin (BCG) is the only vaccine currently recommended and belonging to the World Health Organization (WHO) immunisation schedule in countries with high incidence of TB. It is an attenuated strain obtained in the 1920’s by Calmette and Guérin after 231 passages of a *M. bovis* clinical isolate in glycerinated broth containing bovine bile.^8^ The BCG vaccine is effective in protecting children against severe forms of TB, including tuberculous meningoencephalitis and miliary TB, with greater than 80% efficacy.^8^ ^-^ ^13^ However, protection against pulmonary forms of TB in adolescents and adults varies from 0 to 80%, depending on the study.^14^ ^,^ ^15^ The reason for this variable efficacy is still unknown, but may be associated with several factors, including differences between the BCG strains used in different countries.^16^ ^,^ ^17^ ^,^ ^18^ A BCG booster is used in Brazil for leprosy contacts, family members living near patients. A cohort study showed an increased protective effect of the second dose of the vaccine in leprosy outcomes.^19^

In Brazil, the BCG Moreau strain has been used for vaccine production since the 1920s, but it has only recently been characterised in more detail at the molecular level.^20^ ^,^ ^21^ ^,^ ^22^ ^,^ ^23^ ^,^ ^24^ BCG Pasteur, on the other hand, was the first BCG vaccine strain to have its genome completely sequenced.^25^ It is considered a more recent strain and has been the focus of numerous studies.^26^ The pathogen-host cell interaction is an important aspect impacting vaccine efficacy and given the differences observed between BCG strains. In this context we previously evaluated the impact of the loss of a transcriptional repressor (*rv3405c*) in BCG Moreau in a THP-1 cell infection model,^27^ showing that loss of repression led to a possible functional gain. To expand this scenario, we compared the behaviour of BCG strains Moreau and Pasteur in this macrophage infection model. Our results show that BCG Moreau was better able to survive the hostile intracellular environment, reinforcing the differential physiological behaviour of the Brazilian strain when compared to BCG Pasteur.

## MATERIALS AND METHODS

*Culture of M. bovis BCG* - *M. bovis* BCG Moreau (provided by Fundação Ataulfo de Paiva - FAP) and BCG Pasteur (obtained from the Pasteur Institute, Paris, France) were grown in Middlebrook 7H9 liquid culture medium (DifcoTM) containing Tween 80 (Sigma) and glycerol (Sigma), supplemented with albumin, dextrose and catalase (ADC). The cultivation time was approximately three weeks, under constant agitation at 37ºC, with weekly passages at 1:10 dilution. Upon reaching the optical density (DO600nm) of approximately 1.0, the cultivation was interrupted, aliquoted in 15% glycerol (Sigma) and the seed lot was stored at -80ºC.

To determine bacterial colony forming units (CFU), serial dilutions were made from the bacterial suspension and plated on Middlebrook 7H10 (DifcoTM) supplemented with ADC. Colonies were quantified on the 28th day.

*THP-1 cell culture* - THP-1 cells were cultured in RPMI-1640 medium containing L-glutamine (Sigma), 10% foetal bovine serum (FBS; Sigma) and 0.1% penicillin and streptomycin (PEES; Sigma). Cells were maintained in culture flasks at 37ºC in atmosphere of 5% CO2 by weekly replication.

*Differentiation of THP-1 cells and BCG infection* - For infection assays, THP-1 monocytes were stimulated with 200 nM phorbol 12-myristate 13-acetate (PMA; Sigma) in RPMI-1640 / 10% FBS medium for differentiation in dTHP-1 macrophages. After 48 h of stimulation, the cells were washed to remove PMA and pre-heated RPMI / 10% FBS medium was added for 48 h, with daily change. The THP-1 derived macrophages were infected with *M. bovis* BCG in RPMI / 10% FBS medium at a multiplicity of infection (MOI) of 10: 1 by an infection pulse of 4 h at 37ºC in an atmosphere of 5% CO2. The choice of MOI (10 bacteria per cell) was based on previous studies.^28^ In order to minimise clumping, typical of mycobacteria, bacterial suspensions were passaged 30 times through a 25-gauge needle attached to a 1 mL syringe prior to infection. At the end of the infection time, the culture medium was removed, and the cells were washed twice with RPMI / 10% FBS medium, to remove the bacteria that were not internalised, essentially as described.^29^ ^,^ ^30^

*Intracellular growth of BCG strains* - For the intracellular growth assays, 2 X 10^5^ cells were used per well, in 24-well plates (Nunc), where the cells were differentiated and infected as previously described. Intracellular growth of BCG strains was evaluated through a time kinetics (4 h, 6 h, 24 h, 48 h, 72 h and 96 h) after infection. At the selected times, the medium was removed and the infected THP-1 derived macrophages lysed with 0.05% SDS. The bacterial pellet was resuspended in 1 mL of Middlebrook 7H9 liquid medium containing 10% ADC. Serial dilutions were made from this bacterial suspension and plated on Middlebrook 7H10 as described above. The colonies were quantified on the 28th day and the morphologies documented.

*Co-localisation of bacteria with acidified vesicles* - For the co-localisation assays, 2 X 10^5^ cells were used per well, in 24-well plates (Nunc), where the cells were differentiated on glass coverslips and infected as previously described, with the bacteria previously marked by the fluorophore SYTO9 (LIVE / DEAD^®^ BacLight kit; Molecular Probes - Invitrogen).

After 4 h and 22 h of infection, the acidified vesicles of the infected THP-1 derived macrophages were stained with LysoTracker Red DND-99^®^ fluorophore (Molecular Probes - Invitrogen), following the manufacturer’s instructions. The cells were fixed with 4% paraformaldehyde (PFA) for 20 min at room temperature, protected from light. The nuclei of the eukaryotic cells were stained with 0.5 µg / mL DAPI (Sigma), for 10 min at room temperature and protected from light. Images of 10 random fields were captured using a fluorescence microscope (Nikon Eclipse Ci). At least 250 cells from each studied condition were analysed to observe the percentage of infected cells and 50 isolated bacteria to observe the percentage of bacteria co-localised with acidified phagosomes.^31^ These values were plotted and analysed using the GraphPrism software.

*Quantification of lipid droplets* - The labelling for lipid bodies by oil red O (ORO) was performed at 24 h of infection, essentially as described by Melo et al.^32^ Briefly, cells were fixed with 3.7% formalin for 10 min, washed and incubated in 100% propylene glycol for 5 min followed by incubation with 0.5% ORO for 10 min. Cells were rinsed with 85% propylene glycol for 5 min, followed by water. The nuclei of eukaryotic cells were stained with DAPI (Sigma). Images from 20 random fields were captured totalling a minimum of 250 cells using a fluorescence microscope (Nikon Eclipse Ci). Images were transformed into black and white pictures and analysed with a macro language, kindly provided by Dr Patrícia Bozza (Fiocruz, Brazil), for the open-source image analysis platform Fijl to automatically extract and quantify information from microscopic images. Spots were determined by automatic spot detection and the total area of fluorescent lipid bodies was obtained for each field and divided by the number of cells in the respective field.^33^

*Cytokine assays* - For cytokine dosage assays (IL-1β and MCP-1) 3.2 X 10^4^ cells were used per well, in 96-well plates (Nunc). The cells were differentiated and infected, as described previously. Supernatants from 6 h, 24 h, 48 h, 72 h and 96 h were collected and stored at -80ºC. Using the enzyme-linked immunosorbent assay (ELISA) technique, we determine the concentrations of IL-1β (DY201 - R&D Systems) and MCP-1 (DY279 - R&D Systems), following the manufacturer’s recommendations. The absorbance measurements were obtained on a Molecular Devices microplate reader with a 450 nm filter. The samples were analysed in four biological replicates and the concentration of each cytokine was calculated using the SoftMax program. The data were plotted and analysed using the GraphPrism program.

*Statistical analysis* - Data were expressed as mean ± standard error of the mean (SEM) and statistically analysed using the analysis of variance test (ANOVA), followed by the Bonferroni post-test or the unpaired t-test. All experiments were carried out at least in biological triplicate. The number of replicates is indicated in the caption of the respective figures.

## RESULTS

**Fig. 1:**
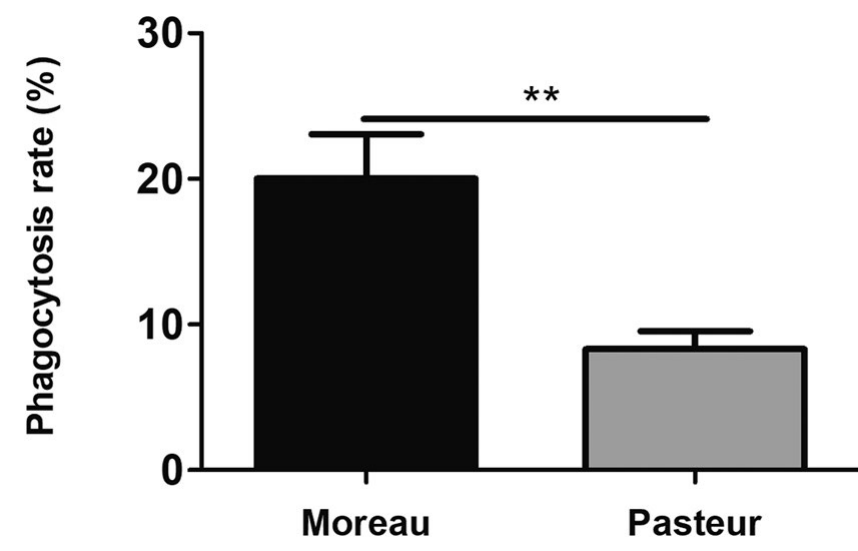
comparison of the internalisation rate of Bacille Calmette-Guérin (BCG) Moreau and Pasteur strains by THP-1 derived macrophages after 4 h of bacteria-host cell interaction. THP-1 monocytes were differentiated into macrophages with 200 nM PMA for 48 h, with subsequent 48-hour rest. THP-1 derived macrophages were infected with the strains studied, using a multiplicity of infection (MOI) 10:1 for 4 h. Colony forming units (CFU) were determined 28 days after plating. Data are presented as mean ± standard error of the mean (SEM). Statistical significance calculated by unpaired t-test **p < 0.01. N = 4.

*Mycobacterium bovis BCG Moreau has a higher internalisation rate in THP-1 derived macrophages* - The internalisation rate for each BCG strain was observed after 4 h of bacteria-host cell interaction. Percentage of internalisation was related to the initial number of bacteria used for infection and common to both strains (2 x 10^6^ bacteria, MOI 10:1). BCG Moreau showed 20% of internalised bacteria, whereas the Pasteur strain showed only 8.5% internalisation (Fig. 1).

**Fig. 2:**
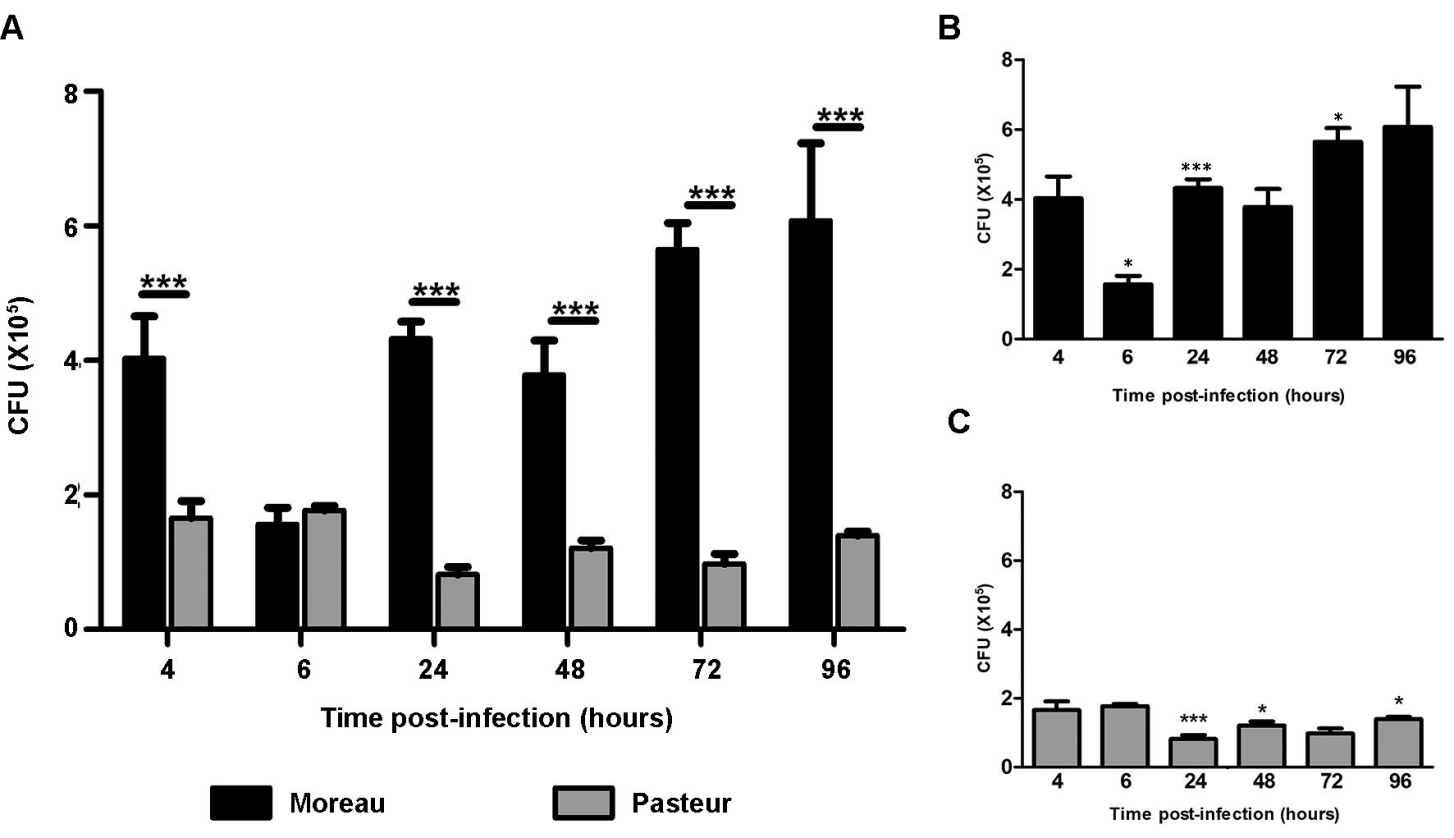
(A) Comparison of the growth profile of Bacille Calmette-Guérin (BCG) strains Moreau and Pasteur in THP-1 derived macrophages. THP-1 monocytes were differentiated into macrophages with 200 nM PMA for 48 h, with subsequent 48-hour rest. THP-1 derived macrophages were infected with the strains studied, using a multiplicity of infection (MOI) 10:1 for 4 h. Colony forming units (CFU) were determined 28 days after plating. Data are presented as mean ± standard error of the mean (SEM). Statistical significance calculated by unpaired t-test ***p < 0.001. N = 4. Growth kinetics of BCG strains Moreau (B) and Pasteur (C) after infection of THP-1 cells. Unpaired t test referring to the previous time * p < 0.05 and *** p < 0.001. N = 4.

*Mycobacterium bovis BCG Moreau has increased numbers of bacteria during infection* - Growth kinetics was assessed from 4 to 96 h post-infection (Fig. 2A). The BCG Moreau intracellular growth profile shows a decrease at 6 h, with a strong and constant increase at subsequent time points. In comparison to 4 h, this decrease observed at 6 h p.i. for the Moreau strain is significant (p < 0.05; Fig. 2B). BCG Pasteur, on the other hand, shows a significant decrease in growth only at 24 h (Fig. 2C) but does not recover growth for the duration of the experiment. The comparison of the intracellular growth profile of these strains showed that BCG Moreau has a higher number of intracellular bacteria compared to BCG Pasteur at all times, except in the 6-hour period. Thus, BCG Moreau is internalised more efficiently than Pasteur, with significantly better intracellular survival up to 96h p.i.

**Fig. 3:**
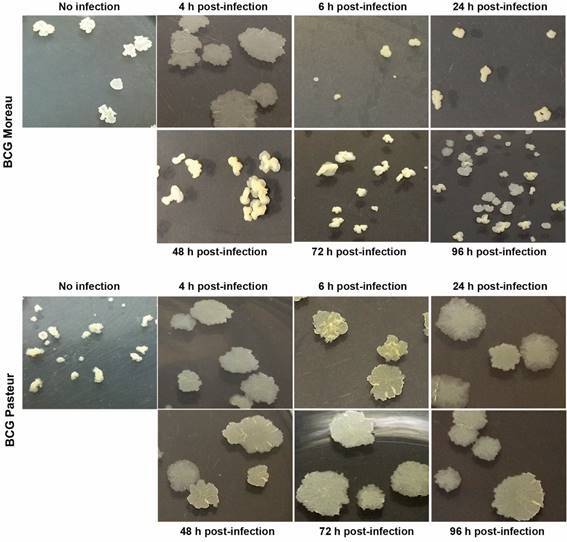
representative profile of Bacille Calmette-Guérin (BCG) Moreau (top panel) and Pasteur (lower panel) colonies after infection of THP-1 derived macrophages. THP-1 monocytes were differentiated into macrophages with 200 nM PMA for 48 h, with subsequent 48-hour rest. THP-1 derived macrophages were infected with the strains studied, using a multiplicity of infection (MOI) 10:1 for 4 h and samples for colony forming units (CFU) counting were removed up to 96 h p.i., as indicated. Images show representative bacterial colonies obtained from lysed macrophages, serially diluted and plated on Middlebrook 7H10, after 28 days. Colonies obtained directly from the serially diluted seed lots prior to infection were used for comparison (no infection). N = 4.

*Mycobacterium bovis BCG Moreau and Pasteur present changes in colony morphology after infection* - The growth of bacteria in 7H10 plates (to determine CFUs) revealed alterations in the colony morphology of the two BCG strains. Fig. 3 shows representative images of BCG Moreau and Pasteur colonies from bacteria obtained from macrophages during time kinetics, compared to the morphology of bacteria obtained from the seed lots (no infection). Within 4 h p.i., both strains present a more spread-out and smooth colony morphology, quite different from the rough and raised colonies observed before infection. Although BCG Moreau colonies readily return to the pre-infection morphology, BCG Pasteur colonies maintain the altered morphology throughout the duration of the experiment. These colony morphology changes were reproducible and observed in all four biological replicates.

**Fig. 4:**
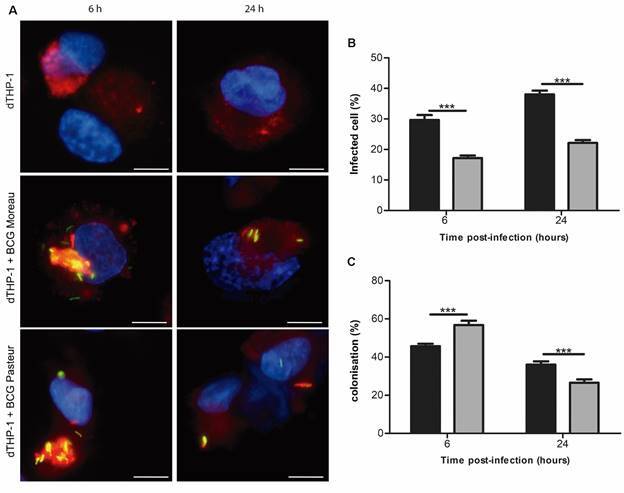
representative co-localisation profile of Bacille Calmette-Guérin (BCG) strains with acidified vesicles at 6 and 24 h p.i. (A) THP-1 monocytes were differentiated into macrophages with 200 nM phorbol 12-myristate 13-acetate (PMA) for 48 h, with subsequent 48-hour rest. Macrophages were infected with the strains studied, using a multiplicity of infection (MOI) 10:1 for 4 h. THP-1 derived macrophages not infected and infected with BCG Moreau or Pasteur were visualised by fluorescence. Nuclei were labelled with DAPI (blue); BCG Moreau and Pasteur labelled with SYTO9 (green); acidified vesicles labelled with LysoTracker (red). Virtual overlay of the images (merge). Scale bar = 10 μm. N = 3. (B) Percentage of THP-1 cells infected with BCG Moreau (black bar) or Pasteur (gray bar) at 6 and 24 h p.i. At least 250 cells were analysed in each condition. (C) Co-localisation of BCG Moreau (black bar) and Pasteur (gray bar) with acidified vesicles at 6 and 24 h At least 50 isolated bacteria were analysed in each condition. Data are presented as mean ± standard error of the mean (SEM). Statistical significance calculated by unpaired t-test ***p < 0.001. N = 3.

*Mycobacterium bovis BCG Moreau phagosomes are more resistant to acidification* - Another parameter evaluated was the co-localisation of the bacteria with acidified vesicles at 6- and 24-h p.i., using LysoTracker staining (Fig. 4A), a measure of phagosome maturation into phagolysosomes. Within 6 h, a greater percentage of cells were infected by BCG Moreau (29%) compared to the BCG Pasteur (17%), increasing to 38% (Moreau) and 26% (Pasteur) at 24 h (Fig. 4B). From the infected cells, we quantified the percentage of co-localisation of Syto-9 labelled bacteria with acidified vesicles (Fig. 4C). At least 50 bacteria distributed in five random fields were analysed by experiment.^31^ ^,^ ^34^ At 6 h, 45% of BCG Moreau and 56% of BCG Pasteur co-localised with acidified vesicles, decreasing to 36% (BCG Moreau) and 26% (BCG Pasteur) at 24 h p.i.

**Fig. 5:**
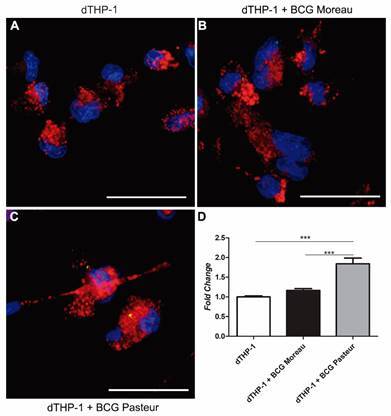
representative profile of the presence of lipid droplet in cells infected with Bacille Calmette-Guérin (BCG) Moreau and Pasteur at 24 h p.i. THP-1 monocytes were differentiated into macrophages with 200 nM phorbol 12-myristate 13-acetate (PMA) for 48 h, with subsequent 48-hour rest. THP-1 derived macrophages were infected with the strains studied, using a multiplicity of infection (MOI) 10:1 for 4 h. THP-1 derived macrophages not infected (A) and infected with BCG Moreau (B) or Pasteur (C). Immunofluorescence detecting the nucleus, labelled with DAPI (blue); BCG Moreau and Pasteur labelled with SYTO9 (green); lipid droplets labelled with oil red O (ORO) (red). Virtual overlay of the images (merge). Scale bar = 50 μm. N = 3. (D) Relative quantification of lipid droplets through ORO staining. At least 250 cells were analysed in each condition. Data are presented as mean ± standard error of the mean (SEM). Statistical significance calculated by analysis of variance (ANOVA) test with Bonferroni post-test *** p < 0.01. N = 3.

*Infection of THP-1 derived macrophages with M. bovis BCG Pasteur induces higher accumulation of lipid droplets* - The accumulation of lipid droplets induced by infection with SYTO9-labelled bacteria was evaluated by ORO staining of infected macrophages at 24 h p.i. (Fig. 5B-C). Quantification shows that the accumulation of lipid droplets in macrophages infected with BCG Pasteur was approximately two times higher when compared to uninfected cells, with only a slight increase (around 1.3 times) when cells were infected with BCG Moreau (Fig. 5D).

**Fig. 6:**
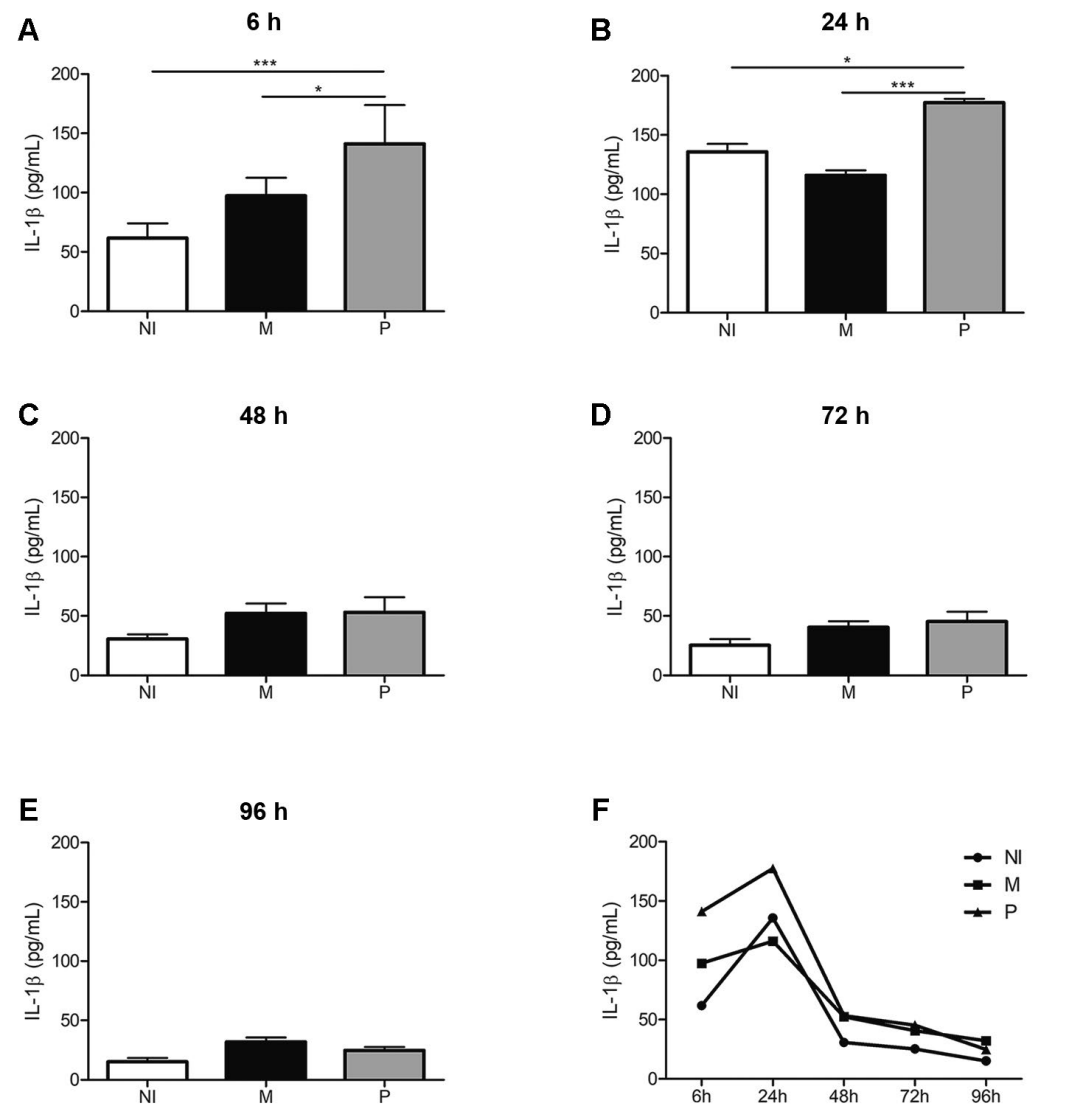
IL-1β secretion profile by THP-1 derived macrophages in response to Bacille Calmette-Guérin (BCG) Moreau (M) and Pasteur (P) infection at 6, 24, 48, 72 and 96 h p.i. (A-E). THP-1 monocytes were differentiated into macrophages with 200 nM phorbol 12-myristate 13-acetate (PMA) for 48 h, with subsequent 48-hour rest. THP-1 derived macrophages were infected with the strains studied, using a multiplicity of infection (MOI) 10: 1 for 4 h. The culture supernatant was collected every 24 h, starting at 6 h. Non-infected THP-1 derived macro-phages (NI) used as controls. Kinetics of secretion profile throughout infection is shown in F. Data are presented as mean ± standard error of the mean (SEM). Statistical significance calculated by analysis of variance (ANOVA) test with Bonferroni post-test *** p < 0.001, * p < 0.05. N = 4.

*Macrophages infected by BCG Pasteur secrete higher levels of IL-1β and MCP-1* - THP-1 derived macrophages infected with BCG Pasteur showed significant higher secretion of IL1-β cytokine at 6 h and 24 h p.i when compared to uninfected cells or to those infected with BCG Moreau (Fig. 6), peaking at 24 h p.i. After 48 h, the secretion of this cytokine falls to levels detected in uninfected macrophages.

**Fig. 7:**
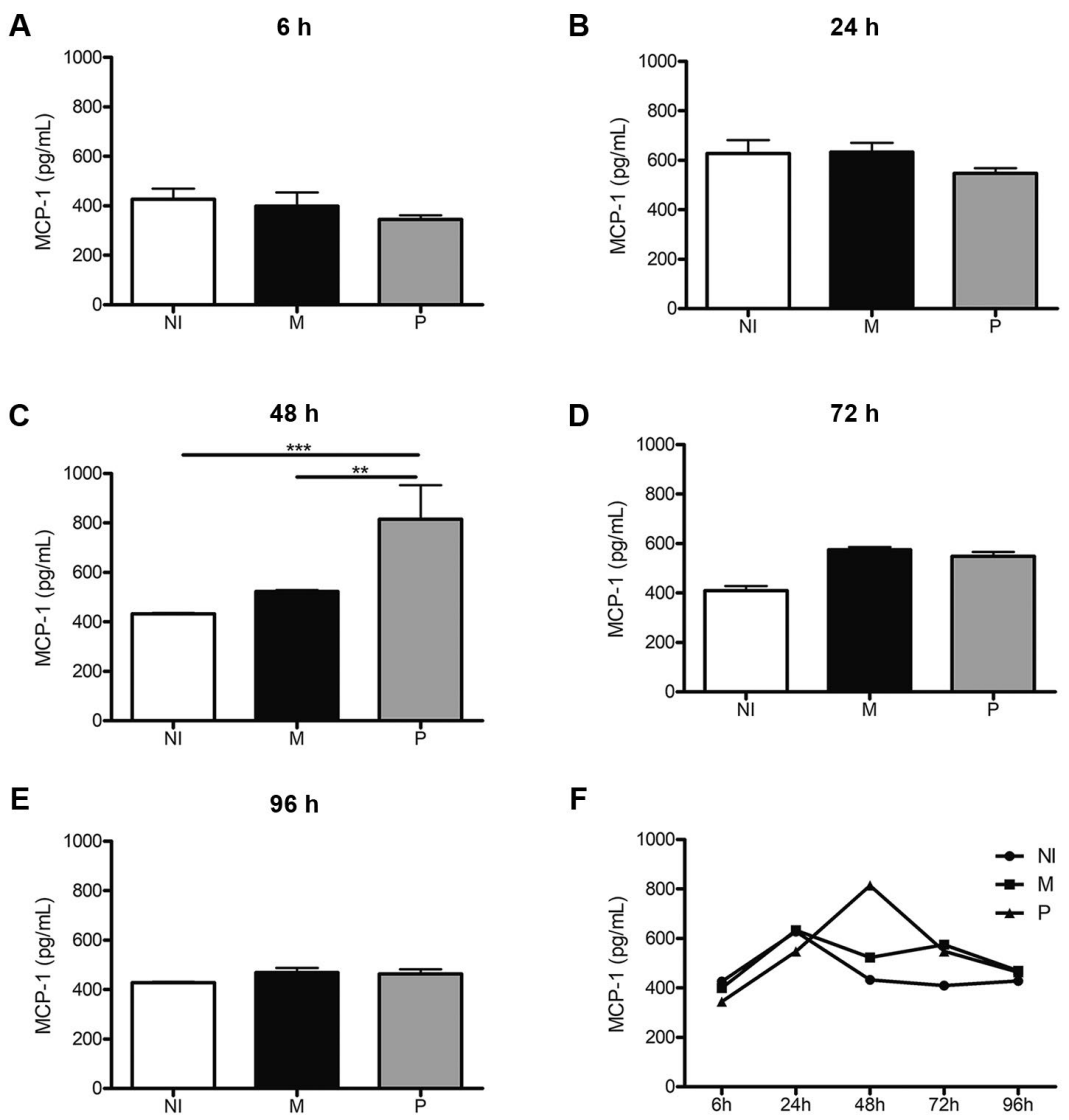
MCP-1 secretion profile by THP-1 derived macrophages in response to Bacille Calmette-Guérin (BCG) Moreau (M) and Pasteur (P) infection at 6, 24, 48, 72 and 96 h p.i. (A-E). THP-1 monocytes were differentiated into macrophages with 200 nM phorbol 12-myristate 13-acetate (PMA) for 48 h, with subsequent 48-hour rest. THP-1 derived macrophages were infected with the strains studied, using a multiplicity of infection (MOI) 10: 1 for 4 h. The culture supernatant was collected every 24 h, starting at 6 hours. Non-infected THP-1 derived macro-phages (NI) used as controls. Kinetics of secretion profile throughout infection is shown in F. Data are presented as mean ± standard error of the mean (SEM). Statistical significance calculated by analysis of variance (ANOVA) test with Bonferroni post-test *** p < 0.001, ** p < 0.01. N = 4.

MCP-1 secretion was also significantly higher in BCG Pasteur infected THP-1 derived macrophages at 48 h p.i, falling to levels detected in uninfected macrophages after 72 h p.i (Fig. 7).

## DISCUSSION

The main purpose of this work was to evaluate the behaviour profile of the Brazilian vaccine strain, BCG Moreau, compared to BCG Pasteur using a THP-1 macrophage model of infection.

Genetic differences between BCG strains have already been characterised and associated with different immunogenic profiles. For example, regarding the two strains chosen for this work, BCG Moreau is deficient in phthiocerol dimycocerosate (PDIM) biosynthesis,^35^ while Pasteur is not, and loss of PDIM has been associated with reduced reactogenicity.^36^ Older strains such as Moreau produce methoxymycolates, strong immunostimulatory molecules, a capacity lost in more recent strains (such as BCG Pasteur).^37^ Differences related to the wall composition, such as the presence of PDIM, can interfere in the binding to receptors, phagocytosis and in the subsequent processes underlying infection.^38^ ^,^ ^39^ ^,^ ^40^ ^,^ ^41^

Both strains were readily taken up by THP-1 derived macrophages after 4 h of bacterial-host cell contact, with markedly different internalisation rates, reflecting the distinct lipid composition of the bacterial wall in each strain. Giovannini et al. demonstrated that a low PDIM composition in H37Rv strains would be sufficient to favour the non-aggregation of bacteria and consequently result in a higher phagocytosis rate.^42^ Therefore, the absence of PDIM in the Moreau strain could be contributing to the increased phagocytosis profile compared to the Pasteur strain.

A comparison of intracellular survival between BCG strains Moreau and Pasteur reveals significantly different profiles. Except for a reduction in viability at 6 h p.i., BCG Moreau showed a constant increase in bacterial numbers up to the final 96 h time point. BCG Pasteur failed to do so. Furthermore, the number of intracellular viable bacteria was always significantly higher for BCG Moreau, except at 6 h p.i. This initial decline may reflect an adaptation period to intracellular stress, but may also result from loss of bacteria due to the possibility that host cells may undergo apoptosis in response to infection;^43^ the removal of medium prior to CFU determination would deplete extracellular bacteria liberated from lysed cells.

The intracellular growth profiles of *M. bovis* and BCG Pasteur in J774 macrophages showed that viability for both strains is reduced in up to 1 day (*M. bovis*) or three days (BCG Pasteur). However, *M. bovis* can revert to a growth phase, while BCG Pasteur maintains constant levels of viable bacteria until the seventh day of infection.^44^ This profile partially reflects what we observed in the THP-1 cell model, where BCG Moreau shows a less attenuated phenotype compared to BCG Pasteur. Indeed, BCG Moreau showed a decrease in viability prior to the Pasteur strain but was capable of regaining growth, while BCG Pasteur maintained the number of intracellular bacilli practically stable until the end of the studied kinetics. This phenomenon may reflect the BCG vaccine genealogy, since the BCG Moreau strain is genetically closer to the original BCG, and consequently to *M. bovis*.^45^

Changes in the colony morphology of bacteria recovered from macrophages were observed while plating to determine CFU counts. Both strains showed morphological changes as early as the first evaluated time (4 h p.i.), from the original small, elevated, rough colonies to a much more spread-out, smooth phenotype. However, while the original morphology was already restored in BCG Moreau at the next evaluated time (6 h p.i.), with few smooth colonies still present, BCG Pasteur colonies remained altered throughout the study period (up to 96 h p.i.). This change in colony morphology probably occurred due to alterations in the lipid composition of the mycobacterial surface, as a form of adaptive plasticity in response to the intracellular environment.^42^ The phenotypic changes in colonies of mycobacteria are observed in *Mtb*, *M. avium*, and *M. abscessus*. The colony’s morphology is highly correlated with the severity of the disease, with the rough morphotype being associated with more severe and persistent infections compared to the smooth morphotype.^42^ ^,^ ^46^ ^,^ ^47^ ^,^ ^48^ In *Mtb* H37Rv strain, the smooth colony phenotype was detected only after passage in monocyte-derived macrophages (MDM), and not in bacteria from axenic culture.^42^ The smooth colony pattern in solid media is associated to attenuated microorganisms, unable to form surface biofilms when grown in liquid media,^49^ and would result from nutrient deprivation and oxidative stress, characterising survival within activated macrophages.^50^ Alteration of the lipid composition of the cell wall and reduction of bacterial multiplication, so that it remains undetectable to the host’s immune system, are adaptive strategies of *Mtb* to survive the adverse intra-phagosomal environment. Bacteria with this smooth colony phenotype would be less virulent, consisting of a population of heterogeneous intracellular bacilli, better able to coexist with the human host. In this way, the host’s immune system would be weakly activated, until favourable conditions were resumed.^42^

In order to gain an initial insight regarding possible differences in the host cell immune response to these two BCG strains, we assayed supernatants for the production of IL-1β and MCP-1. These were chosen due to their known role in control of bacterial load (IL-1β) and ROS response (MCP-1)^51^ in the context of BCG infected THP-1 derived macrophages. The secretion of the cytokine interleukin-1β (IL-1β) that controls bacterial load through the recruitment of microbial effectors^52^ was assessed by ELISA in the supernatant of infected THP-1 derived macrophages. An increase in its concentration was observed at 6 h p.i., reaching a peak at 24 h, with a tendency to return to levels like those of uninfected macrophages at other times. This 24-hour peak of expression can lead to the induction of antimicrobial effectors, such as tumour necrosis factor (TNF), to contain the infection. TNF would then trigger the apoptosis mechanism, which would lead to decreased viability in macrophages and bacilli.^43^ ^,^ ^53^ In addition, the intracellular bacillary load remained low and constant, suggesting that the microbicidal mechanism triggered by IL-1β would be affecting the viability of the BCG Pasteur strain. However, for cells infected with BCG Moreau, the increase in the secretion of the cytokine IL-1β was less pronounced, perhaps insufficient to generate a response in the same proportion as that observed for BCG Pasteur.

The increased secretion of the chemokine MCP-1 (CCL2) observed in macrophages infected with BCG Pasteur at 48 h p.i. but not with BCG Moreau, may be reflecting an increased immune response against infection with this strain. The induced secretion of chemokines CCL2, CCL3, CCL4 and CCL5 has already been observed in THP-1 and PBMC-derived macrophages when infected with BCG.^51^ ^,^ ^54^ *M. bovis* BCG infection induces the intracellular formation of ROS, generated by nicotinamide adenine dinucleotide phosphate (NADPH) oxidase. The accumulation of ROS plays an important role as a signalling molecule in the regulation of CCL2 chemokine secretion, contributing to the molecular response in the signalling of the inflammatory process.^51^ Thus, in a similar way, we could infer that the BCG Pasteur strain would be inducing a greater formation of reactive oxygen species, which would be neutralised due to the higher production of antioxidants in this strain when compared to the more primitive strains such as BCG Moreau.^45^ These preliminary results should be further explored, expanding the analysis of host molecules differentially expressed in response to the infection with different BCG strains.

Fluorescence analyses of infected THP-1 derived macrophages with the labelled BCG strains indicated a higher percentage of cells infected with the BCG Moreau strain in 6 and 24 h, suggesting that in 6 hours there are less bacilli per cell in macrophages infected by BCG Moreau, since the number of viable bacilli was similar when compared to the BCG Pasteur strain. The possible reinfection of cells by extracellular bacteria liberated earlier in infection^43^ could contribute to the increase in the percentage of infected cells at subsequent time points. The higher rate of co-localisation of cells infected with BCG Pasteur in 6 h suggests that these bacilli are failing to successfully evade microbicidal mechanisms and are likely to be eliminated. In comparison, co-localisation (%) for BCG Moreau was less variable between these two time points.

The survival of pathogenic mycobacteria within a phagosome is based on the bacterium’s ability to control phagosomal maturation differently from non-pathogenic. J774 cells infected with BCG Pasteur for 3 h show about 40% of the bacilli co-localised with acidified vesicles and a decrease in the intracellular viability at 24 h p.i.,^55^ corroborating with our data.

Macrophage infection with bacteria also triggers the modulation of lipids, which favour the escape of the pathogen.^56^ The accumulation of lipid droplets (LDs) in macrophages infected by *M. bovis* BCG was associated with Toll-Like Receptor-2 (TLR-2), with the modulation of cytokines IL-10 and TNF-α, as well as the presence of prostaglandin E2 (PGE2).^7^ The use of fatty acids as a carbon source by *Mtb* during infection is widely recognised.^57^ Mycobacteria can store fatty acids in the form of triacylglycerol, detected during dormancy^58^ ^,^ ^59^ and also in the sputum of TB patients.^60^ ^,^ ^61^ The use of this triacylglycerol occurs when *Mtb* is deprived of nutrients^62^ and when BCG is reactivated after a non-replicative state during hypoxia,^63^ showing an important role of intracellular lipid storage during the non-replicative phase and in reactivation.^64^ Thus, the greater accumulation of LDs observed in macrophages infected by BCG Pasteur could favour its persistence within the intra-phagosomal environment, since the formation of LD is correlated with the inhibition of autophagy and phagosomal acidification,^65^ possibly suggesting that BCG Pasteur is in a viable but non-replicative state.

Overall, our results show that, compared to Pasteur, BCG Moreau can infect more efficiently and grow steadily in THP-1-derived macrophages, important characteristics in the development of an efficient immune memory by a live vaccine.^66^ ^,^ ^67^ The observed lower production of ILβ-1b and MCP-1 by BCG Moreau infected macrophages point in the direction of a reduced capacity, by this strain, to signal a bactericidal response. These data should be further expanded in order to allow correlations to the vaccine’s protective efficacy, but already indicate important differences between the two strains.
